# Supplementary material for: APRICOT: an integrated computational pipeline for the sequence-based identification and characterization of RNA-binding proteins
Source: Nucleic Acids Res. 2017 Mar 2;45(11):e96. doi: 10.1093/nar/gkx137 (PMC5499795; doi:10.1093/nar/gkx137)
Supplement: Supplementary Data [file gkx137_supp.zip › nar-01420-met-n-2016-File006.pdf]

Legends for the supplementary materials

Supplementary Material S1. (A) Different methods in APRICOT involved in the feature based scoring of the predicted domains. (B) Different modules of APRICOT for the additional annotations of selected proteins.

Supplementary Figure S2. Resulting table obtained upon the analysis of the PTBP1 by APRICOT. The columns in the table have been explained in three parts: (A) annotation of the protein retrieved from UniProt knowledgebase, (B) annotation of the domains in the protein selected by APRICOT, and (C) statistical output for each parameter associated with the domain predictions.

Supplementary Table S3. Description of different RBP and non-RBP datasets used in this study for training and evaluation of APRICOT pipeline.

Supplementary Table S4. Analysis of the training sets used in this study. (A) SwissProt-positive and SwissProt-negative protein datasets selected from SwissProt (UniProt knowledgebase) by APRICOT using different parameters. (B) The values for the different assessment measures (SN, SP, ACC, MCC and *F* measure) calculated for SwissProt-positive and SwissProt-negative at systematically varying cut-offs for different parameters associated with domain predictions.

Supplementary Figure S5. The Receiver Operating Characteristic (ROC) curves and their Area Under Curves (AUC) for each parameter and combinations of parameters to assess their marginal contributions to the overall accuracy of APRICOT with which it identifies RBPs.

Supplementary Table S6. Overview of RBDs used in this study and results obtained from APRICOT analysis of the human RBPs selected from the experimental studies. (A) List of known RBD families from different benchmark studies. (B) *Reference domain set* for RBDs used in this study. (C) List of human RBPs retrieved from different studies. (D) APRICOT based identification of RBPs in human demonstrating high accuracy of the pipeline.

Supplementary Table S7. Results obtained from APRICOT analysis of the RBPs from *E. coli* (strain K-12). (A) List of of E.coli RBPs selected from the experimental studies and extended by proteins selected from UniProt database using the Gene Ontology term for RNA-binding (GO:0003723). (B) Results obtained from APRICOT analysis of the RBPs from *E. coli* (strain K-12). (C) Summary of RBPs identified by APRICOT in *E.coli* using *Reference domain set* listed in S6 (B).

Supplementary Table S8. Identification and characterization of kinase proteins in *E. coli* (strain K-12) using APRICOT. (A) Data set of kinase proteins from *E. coli* (strain K12) selected from SwissProt. (B) APRICOT based identification of kinases from the reference set of *E. coli* kinase proteins.

Supplementary Table S9. Table showing a synopsis of NBench dataset analyzed by APRICOT, which demonstrates the potential of complementing APRICOT with the tools for the prediction of RNA-binding sites. (A) RNA-binding residues of 3657 proteins in Nbench_3657 datasets and their overlap with the domain sites as predicted by APRICOT. (B) Description of RBPs that were correctly identified as RBPs from Nbench_3657 by APRIOCT pipeline.
