# Supplementary material for: APRICOT: an integrated computational pipeline for the sequence-based identification and characterization of RNA-binding proteins
Source: Nucleic Acids Res. 2017 Mar 2;45(11):e96. doi: 10.1093/nar/gkx137 (PMC5499795; doi:10.1093/nar/gkx137)
Supplement: Supplementary Data [file gkx137_supp.zip › nar-01420-met-n-2016-File007.pdf]

## S1 (A) Methods involved in feature-based scoring of the predicted domains

**1. Chemical properties:** The chemical properties help in determining biological aspects like catalytic properties in living cells, involvement in cellular processes, three-dimensional folding and the structure stability. To include these aspects into the APRICOT analysis, we calculate the similarity between the region of predicted domains in the query proteins and their corresponding fragments in the references by means of chemical-properties, namely for average mass, pKa values and isoelectric point (pI)[1-6]. The values for each feature in the predicted domains are divided by the values of corresponding feature in the reference domains and a score in the range of 0 to 1 are obtained suggesting the extent of functional similarity in the predicted domains. This analysis is based on the assumption that the high conservation in the predicted domain compared to its reference will result in comparable chemical properties.

**2. Needleman-Wunsch global alignment scores:** To calculate the extent of similarity between the predicted domain region in the query and its corresponding reference sequence, APRICOT carries out their global alignments using Needleman-Wunsch algorithm[7] implemented in Biopython[8]. This algorithm uses dynamic programming to compare biological sequences and uses match scores and gap penalties, however in APRICOT we did not introduce any gap penalty. The similarity scores are calculated for the global alignments of two sequence features: primary amino acid sequence and secondary structure. The similarity scores between the query and reference sequences are obtained that range from 0 to 1, where 1 is a complete match.

**3. Euclidean distances of protein compositions:** A protein composition refers to the fraction of each amino acid group (di-peptides, tri-peptides) or properties (physico-chemical or secondary structure) within a protein. It has been shown that function-specific information, for example subcellular localization, secondary structure, enzyme families, and membrane protein types can be indicated by such compositions[9-14] Therefore, the similarity in compositions between the predicted domains and their corresponding references can reflect the functional significance of the predictions and therefore inform the user of the putative biological function conferred by the identified domain. These similarities are calculated by

Euclidean distance, and the similarity score (1-Euclidean distance) is represented in a range of 0 to 1, where 1 stands for an absolute match.

**4. Homology between predicted sites and reference domains:** The last set of properties considered for feature-based scoring is the homology by means of similarity, identity, gaps and coverage obtained for the predicted domain sites in the query with respect to the sites in their reference domains. The domain coverage is calculated by dividing the residue counts of the predicted domain site in the query protein by the original length of the reference domain. The similarity, identity and gap are calculated by dividing the corresponding residue counts in the predicted domain by the calculated domain coverage (rather than the full length of the domain). Each of these parameters is reported in a value range of 0 to 1. The coverage value of 1 indicates an identification of a complete domain in the query. The similarity and identity value of 1 indicates an absolute match in the fraction of domain identified in the query. The gap value of 0 means no gap in the sequence, which is represented as the measure of 1-gap so that a score closer to 1 represents a favourable scenario.

## S1 (B) Modules for the additional annotations of selected proteins

**1. Identification sub-cellular localization of the proteins:** Information about the sub-cellular localization can assist in deriving the potential functional role associated with a protein. A standalone version of PSORTb v.3.3[15] is used for computational prediction of the subcellular localization of selected proteins. PSORTb provides a list of five localization sites (cytoplasmic, cytoplasmic membrane, cell wall, extracellular and secondary localization) and the associated probability score (0-10 indicating low to high probability).

**2. Secondary structure calculation by RaptorX:** In principle an amino-acid sequence that aligns well with annotated proteins could be considered as functional homologs. However, amino acid conservation at the sequence level is not always obvious when dealing with the sequences where only functional domains are conserved whereas rest of the sequence share structure homology. In such cases, the selection of true homologs based on primary sequences is difficult. To address this problem, the candidate proteins can be compared to the known proteins at the structural level. The structure prediction tool RaptorX[16] has been

integrated in the pipeline for the prediction of protein secondary structures. For example, two-dimensional structure information complemented with the domain prediction can be used for characterizing the affinity of a protein for RNA. It is also possible to derive tertiary structure without close homologs in the Protein Data Bank[17] (PDB) using RaptorX, allowing further functional characterization.

**3. Tertiary structure homologs from Protein Data Bank (PDB):** The tertiary structures are critical to identify the ligand partners of proteins in order to achieve a high-resolution annotation. Out of several millions of proteins available in non-redundant (nr) database in NCBI[18], only 105,417 proteins and 5,198 protein/nucleic-acid complexes (November 2015) have been crystalized. There are numerous computational tools available for the estimation of tertiary structures of proteins, e.g. PHYRE2[19], CPHModels[20] and I-TASSER online[21]. Most of these methods are computationally demanding and are available only as web-servers making their integration difficult into automated workflows. As such, in order to provide a quick insight into the potential binding mechanisms of selected proteins based on the available annotation of the PDB structure homologs, APRICOT lists known tertiary structure homologs for the query proteins from PDB.

**4. Gene Ontology:** The Gene Ontology or GO consortium[22] is a bioinformatics initiative for unifying annotation by means of controlled vocabulary. GO terms are widely used for standard annotation of a gene with various information, including cellular localization, biological processes, and molecular function. GO is determined by extracting all GO terms available for a protein in UniProt database[23] and for the domains in InterPro and CDD databases. In order to achieve a broader GO catalogue for each candidate protein, Blast2GO[24] can be executed from APRICOT subcommand blast2go when already installed by the users.

## References

1. A. A. Zamyatin, Prog. Biophys. Mol. Biol., 24(1972)107-123

2. C. Chothia, J. Mol. Biol., 105(1975)1-14

3. C. Tanford, Adv. Prot. Chem., 17(1962)69-165

4. P.J. Linstrom and W.G. Mallard, Eds., NIST Chemistry WebBook, NIST Standard Reference Database Number 69, National Institute of Standards and Technology, Gaithersburg MD, 20899, http://webbook.nist.gov, (retrieved April 14, 2016).

5. Structural Biochemistry/Proteins/Structures. (2016, April 7). Wikibooks, The Free Textbook Project. Retrieved 14:35, April 14, 2016 from https://en.wikibooks.org/w/index.php?title=Structural_Biochemistry/Proteins/Structures&oldid=3069652.

6. The Merck Index, Merck & Co. Inc., Nahway, N.J., 11(1989); CRC Handbook of Chem.& Phys., Cleveland, Ohio, 58(1977)

7. Needleman, S. B., and Wunsch, C. D. (1970) "A General Method Applicable To The Search For Similarities In The Amino Acid Sequence Of Two Proteins". Journal of Molecular Biology 48.3 (1970): 443-453. Web.

8. Chapman, B. and Chang, J. (2000) "Biopython". SIGBIO Newsl. 20.2: 15-19. Web.

9. Shen, H. and Chou, K. (2008) PseAAC: A flexible web server for generating various kinds of protein pseudo amino acid composition. Analytical Biochemistry, 373, 386-388.

10. Reczko, M. and Hatzigeorgiou, A. (2004) Prediction of the subcellular localization of eukaryotic proteins using sequence signals and composition. Proteomics, 4, 1591-1596.46. Romeo, T. (1998) Global regulation by the small RNA-binding protein CsrA and the non-coding RNA molecule CsrB. *Molecular Microbiology*, 29, 1321-1330.

11. Habib, T., Zhang, C., Yang, J., Yang, M. and Deng, Y. (2008) Supervised learning method for the prediction of subcellular localization of proteins using amino acid and amino acid pair composition. BMC Genomics, 9, S16.

12. Eisenhaber, F., Frömmel, C. and Argos, P. (1996) Prediction of secondary structural content of proteins from their amino acid composition alone. II. The paradox with secondary structural class. Proteins: Structure, Function, and Genetics, 25, 169-179.

13. Otaki, J., Tsutsumi, M., Gotoh, T. and Yamamoto, H. (2010) Secondary Structure Characterization Based on Amino Acid Composition and Availability in Proteins. Journal of Chemical Information and Modeling, 50, 690-700.

14. Cai, Y. and Chou, K. (2006) Predicting membrane protein type by functional domain composition and pseudo-amino acid composition. Journal of Theoretical Biology, 238, 395-400.

15. Yu, N., Wagner, J., Laird, M., Melli, G., Rey, S., Lo, R., Dao, P., Sahinalp, S., Ester, M. and Foster, L. et al. (2010) PSORTb 3.0: improved protein subcellular localization prediction with refined localization subcategories and predictive capabilities for all prokaryotes. Bioinformatics, 26, 1608-1615.

16. Källberg, M., Wang, H., Wang, S., Peng, J., Wang, Z., Lu, H. and Xu, J. (2012) Template-based protein structure modeling using the RaptorX web server. Nat Protoc, 7, 1511-1522.

17. Kouranov, A. (2006) The RCSB PDB information portal for structural genomics. Nucleic Acids Research, 34, D302-D305.

18. Pruitt, K. D., Tatusova, T., & Maglott, D. R. (2005). NCBI Reference Sequence (RefSeq): a curated non-redundant sequence database of genomes, transcripts and proteins. Nucleic Acids Research, 33(Database Issue), D501–D504.

19. Kelley, L., Mezulis, S., Yates, C., Wass, M. and Sternberg, M. (2015) The Phyre2 web portal for protein modeling, prediction and analysis. Nat Protoc, 10, 845-858.

20. Nielsen, M., Lundegaard, C., Lund, O. and Petersen, T. (2010) CPHmodels-3.0--remote homology modeling using structure-guided sequence profiles. Nucleic Acids Research, 38, W576-W581.

21. Zhang, Y. (2008) I-TASSER server for protein 3D structure prediction. *BMC Bioinformatics*, 9, 40.

22. Gaudet, P. (2009) The GO's Reference Genome Project: A Unified Framework for Functional Annotation across Species. *PLoS Comput Biol*, 5, e1000431.

23. Magrane, M., and Consortium, U. (2010) UniProt Knowledgebase: a hub of integrated data. *Nature Precedings*, 10.1038/npre.2010.5092.

24. Conesa, A. and Götz, S. (2008) Blast2GO: A Comprehensive Suite for Functional Analysis in Plant Genomics. *International Journal of Plant Genomics*, 2008, 1-12.
