# Supplementary material for: APRICOT: an integrated computational pipeline for the sequence-based identification and characterization of RNA-binding proteins
Source: Nucleic Acids Res. 2017 Mar 2;45(11):e96. doi: 10.1093/nar/gkx137 (PMC5499795; doi:10.1093/nar/gkx137)
Supplement: Supplementary Data [file gkx137_supp.zip › nar-01420-met-n-2016-File008.pdf]

S2. A snapshot of HTML table generated by APRICOT to list the resulting RBPs with statistical values of different parameters and annotations, please see the video tutorial (Part-9) as well for the explanation.

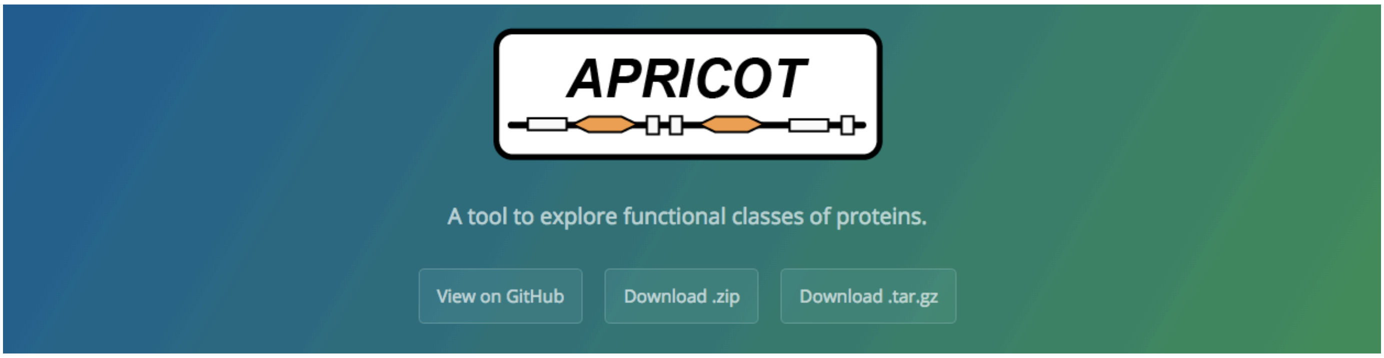


S2 (A) The annotation of proteins retrieved from UniProt Knowledgebase which lists information like length of protein, gene names, Gene Ontology, cross-reference to EMBL, PDB, KEGG etc.

Show 10 entries

| Entry | Entry name | Protein names | Organism | Length | Gene names |
|---|---|---|---|---|---|
| P26599 | PTBP1_HUMAN | Polypyrimidine tract-binding protein 1 (PTB) (57 kDa RNA-binding protein PPTB-1) (Heterogeneous nuclear ribonucleoprotein I) (hnRNP I) | Homo sapiens (Human) | 531 | PTBP1 PTB |
| P26599 | PTBP1_HUMAN | Polypyrimidine tract-binding protein 1 (PTB) (57 kDa RNA-binding protein PPTB-1) (Heterogeneous nuclear ribonucleoprotein I) (hnRNP I) | Homo sapiens (Human) | 531 | PTBP1 PTB |

Search:

| Existance-Type | GO | EMBL-ID | PDB-ID | KEGG-ID |
|---|---|---|---|---|
| evidence at protein level | 'GO:0070062->C:extracellular exosome', 'GO:0016020->C:membrane', 'GO:0005730->C:nucleolus', 'GO:0005654->C:nucleoplasm', 'GO:0000166->F:nucleotide binding', 'GO:0044822->F:poly(A) RNA binding', 'GO:0008380->P:RNA splicing' | X62006 | 1QM9 | hsa:5725 |
| evidence at protein level | 'GO:0070062->C:extracellular exosome', 'GO:0016020->C:membrane', 'GO:0005730->C:nucleolus', 'GO:0005654->C:nucleoplasm', 'GO:0000166->F:nucleotide binding', 'GO:0044822->F:poly(A) RNA binding', 'GO:0008380->P:RNA splicing' | X62006 | 1QM9 | hsa:5725 |

S2 (B) The annotation of the domain entries selected by APRICOT consisting of the information like resource of domain entry, domain id, name, the user-provided term that selected this domain, another member in the domain database with similar motif, length of domain, the start and end location in the query protein indicating the region where the domain was detected.

| Resource | ResourceID | DomainID | ShortName | FullName | DomainKeyword |
|---|---|---|---|---|---|
| CDD | 249565 | PF00076 | RRM_1 | 1 RNA recognition motif. (a.k.a. RRM, RBD, or RNP domain). The RRM motif is probably diagnostic of an RNA binding protein. | RNA-bind, RRM |
| CDD | 273733 | TIGR01649 | HnRNP-L/PTB/hephaestus splicing factor | Included in this family of heterogeneous ribonucleoproteins are PTB (polypyrimidine tract binding protein ) and hnRNP-L. These proteins contain four RNA recognition motifs. | RNA-bind |

| DomainGo | Members | DomainLength | Start | Stop |
|---|---|---|---|---|
| mf:GO:0003676 [nucleic acid binding] | NA | 70 | 458 | 517 |
| mf:GO:0003723 [RNA binding],bp:GO:0006397 [mRNA processing],cc:GO:0005634 [nucleus] | NA | 481 | 57 | 531 |

S2 (C) The statistical result of the domain predicted by APRICOT analysis, comprising of the values for different parameters like E-values, bit-scores, domain coverage, residue identity, sequence similarity, gap and their values in percentage. It also tags each domain entries with 'ParameterSelected' if it passes or 'ParameterDiscarded' if it does not pass the parameter cut-offs (default or if defined by the users).

| E-value | BitScore | Bits | DomainCoverage | CoveragePercent | Identity | IdentityPercent |
|---|---|---|---|---|---|---|
| 0.001 | 38.4 bits (90) | 38.4 | 59 | 84.2857 | 20/63 (31%) | 28.5714 |
| 0.0 | 648 bits (1674) | 648 | 474 | 98.5447 | 254/515 (49%) | 52.8067 |

| Similarity | SimilarityPercent | Gaps | GapPercent | ParameterFilterTag |
|---|---|---|---|---|
| 32/63 (50%) | 45.7143 | 3/63 (4%) | 4.2857 | ParameterSelected |
| 315/515 (61%) | 65.4886 | 74/515 (14%) | 15.3846 | ParameterSelected |
