# Supplementary material for: APRICOT: an integrated computational pipeline for the sequence-based identification and characterization of RNA-binding proteins
Source: Nucleic Acids Res. 2017 Mar 2;45(11):e96. doi: 10.1093/nar/gkx137 (PMC5499795; doi:10.1093/nar/gkx137)
Supplement: Supplementary Data [file gkx137_supp.zip › nar-01420-met-n-2016-File011.pdf]

Sharan *et al.*, 2016, Supplementary Figure S5

The Receiver Operating Characteristic (ROC) curves and their Area Under Curves (AUC) for each parameter and combinations of parameters to assess their marginal contributions to the overall accuracy of APRICOT with which it identifies RNA-binding proteins.

The decision values of the parameters were evaluated for all the predicted RNA-binding domain entries in the training sets. Subsequently, the ROC and AUC were generated for the ranking of the parameters using the module "ROC Curve for Binary SVM" (authors: Tingfan Wu, Chien-Chih Wang, and Hsiang-Fu Yu) from the LIBSVM Version 3.21, a package for support vector machines (Chih-Chung Chang and Chih-Jen Lin, 2015). As shown below, the combination of sequence similarity and domain coverage showed the best AUC (0.91), hence, they were selected as default parameters for APRICOT analysis.

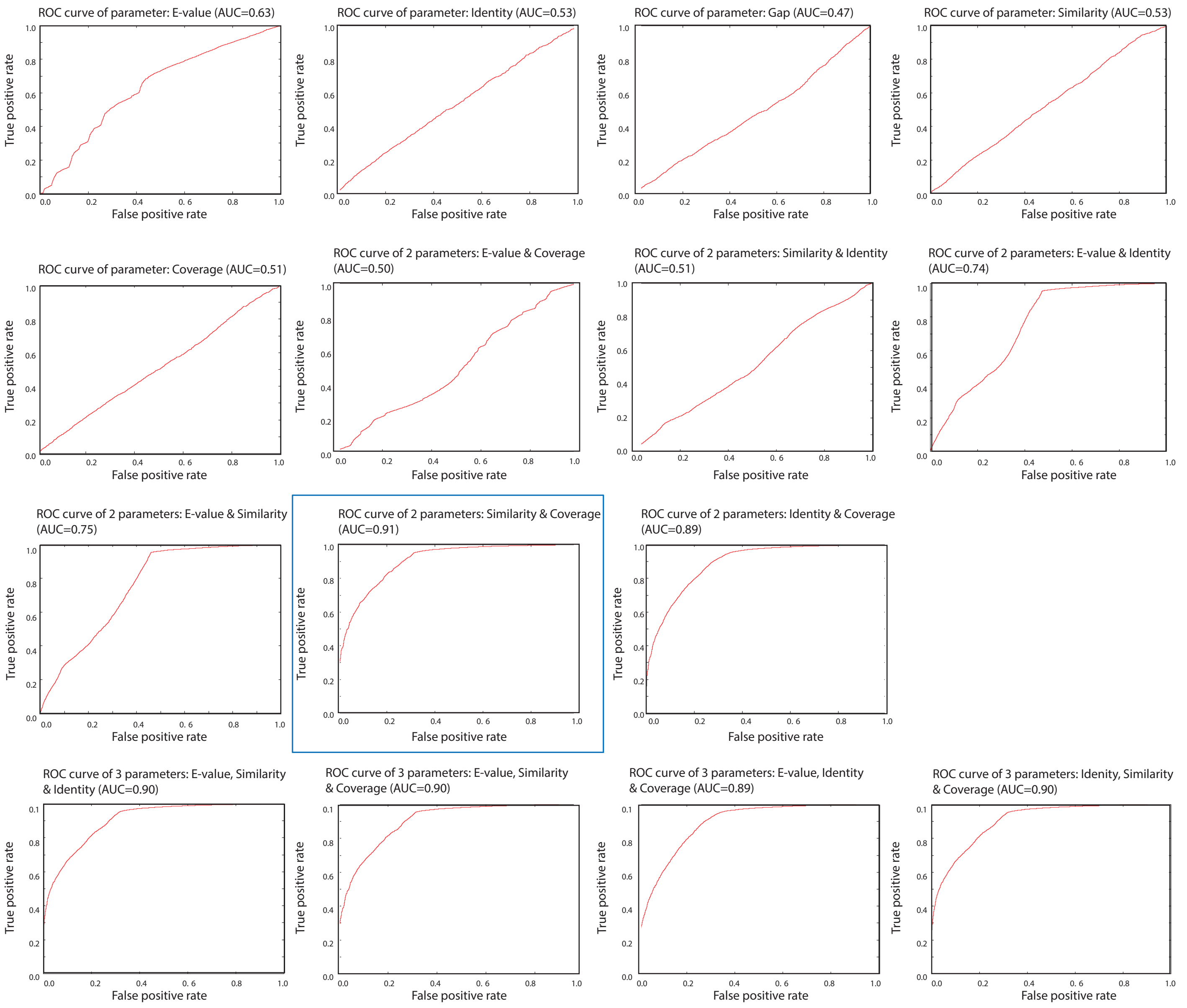

Reference:

Chih-Chung Chang and Chih-Jen Lin, LIBSVM : a library for support vector machines. ACM Transactions on Intelligent Systems and Technology, 2:27:1--27:27, 2011. Software available at http://www.csie.ntu.edu.tw/~cjlin/libsvm
